# Correction to *Lancet Psychiatry* 2020; published online Nov 9. https://doi.org/10.1016/S2215-0366(20)30462-4

**DOI:** 10.1016/S2215-0366(20)30509-5

**Published:** 2021-01

*Taquet M, Luciano S, Geddes J R, Harrison P J. Bidirectional associations between COVID-19 and psychiatric disorder: retrospective cohort studies of 62 354 COVID-19 cases in the USA.* Lancet Psychiatry *2020; published online Nov 9. https://doi.org/10.1016/S2215-0366(20)30462-4*—In this Article, the y axes in figure 2 were labelled incorrectly. These corrections have been made to the online version as of Nov 12, 2020, and will be made to the printed version.

